# Characterization of Cell–Surface Interactions of Ligands Using ^19^F NMR and DNP Hyperpolarization

**DOI:** 10.1021/acs.analchem.5c04644

**Published:** 2025-12-26

**Authors:** Chang Qi, Nirmalya Pradhan, Christian Hilty

**Affiliations:** Chemistry Department, Texas A&M University, College Station, Texas 77843, United States

## Abstract

The binding of small molecule ligands to membrane lipids and membrane proteins in live cells is characterized by their effect on nuclear spin relaxation. The hyperpolarization of ^19^F spins provides signal enhancements for the measurement of *R*_2_ relaxation in single-scan NMR experiments. The relaxation rates of two prototype drug molecules, 2-methyl-3-(5-methylsulfanyl-[1,3,4]­oxadiazol-2-yl)-6-trifluoromethyl-pyridine (ICT5040) and 4-(trifluoromethyl)­benzene-1-carboximidamide (TFBC), are found to increase linearly with cell density in the presence of several different cell types. The linear increase is interpreted as the initial slope of a binding saturation curve. A stronger relaxivity of cells toward the hyperpolarized ITC5040 is observed compared to small unilamellar vesicles, when normalized to lipid concentration in each case. The difference in relaxation rates is not fully accounted for by the size difference between cells and vesicles. A larger effect of the suspension-grown DU4475 human breast cancer cells compared to adherent 4T1 mouse breast cancer cells and HEK293T human embryonic kidney cells is observed. This difference is reduced when DU4475 cells are subjected to the same trypsin treatment that is required for adherent cells, supporting the conclusion that the interaction with proteins increases the relaxivity of the cells toward the ligand molecules. The influence of these parameters suggests that model membranes are not always sufficient for binding studies, and that hyperpolarized *R*_2_ relaxometry may be used for determining the interactions of ligand molecules with cells in biophysical studies or for drug discovery.

## Graphical abstract

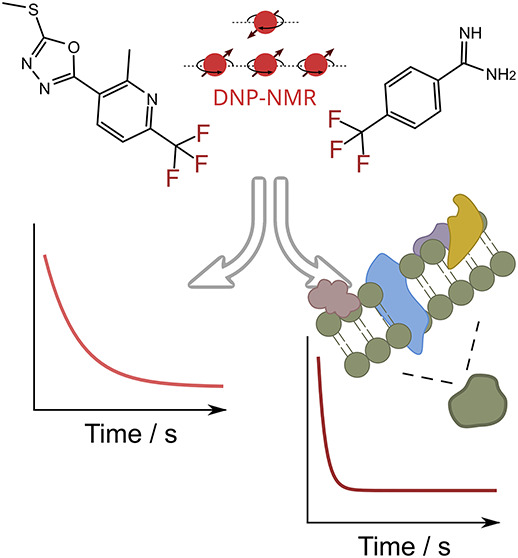

## Introduction

The interactions between small molecules and the cell membrane, as well as diverse proteins in the membrane bilayer, modulate basic cellular functions such as signaling and metabolic processes. Likewise, the function of cellular compartments is strongly dependent on the internal membranes. Measurements of the binding of ligands to membranes and membrane proteins underpin the understanding of basic biological processes and are important in the discovery of new drugs.^1^ Because of the complexity of cell membranes, biomimetic lipid membrane systems including soluble vesicles and solid-supported lipid bilayers have been designed as simplified models for cellular membranes.^2^ Although the composition and size of biomimetic lipid membranes are flexible and easy to control, these membranes necessarily contain fewer varieties of proteins than cells and lack biological functions. The behavior of molecular species that interact with the membrane of living cells may differ from those of these models, blurring the distinction of physiological functions from multiple cell types. Analytical methods that allow for the detection of ligand binding to the membrane of live cells are required to differentiate these properties. Fluorescence-based methods,^3^ including fluorescence correlation spectroscopy (FCS) and fluorescence activated cell sorting (FACS), and NMR approaches that focus on ligand observation, including saturation transfer difference (STD),^4,5^ water-ligand observed via gradient spectroscopy (WaterLOGSY),^6^ as well as transferred nuclear Overhauser effect spectroscopy (trNOESY),^7^ have been applied for this purpose.

Dissolution dynamic nuclear polarization (D-DNP) facilitates the NMR measurement of minor species in liquids by boosting the observable signal.^8^ The D-DNP technique has been demonstrated to allow the observation of ligand interactions with macromolecules.^9–12^ Transiently measured NOEs can provide information on molecular contacts to determine ligand binding affinity and the conformation of bound ligands,^13–15^ and even the interaction of biological molecules with membranes.^16^ While the cross-relaxation rates that are the basis for the NOE are directly indicative of molecular structure and contacts, ligand binding can also be inferred from other relaxation-derived parameters measured using D-DNP hyperpolarization.^17^ The spin–spin relaxation rate (*R*_2_) is highly sensitive to a change in molecular motion, enabling measurement of the binding of a DNP hyperpolarized ligand to a protein in a single scan. Changes in *R*_2_ relaxation rates of a hyperpolarized molecule that competes with another ligand for the same binding pocket can be used to determine the binding affinity.^18^ Although *R*_2_ relaxation does not contain direct information on molecular contacts, relaxation dispersion on the basis of chemical shift differences between free and bound forms can be combined with D-DNP to acquire binding kinetic parameters and characterize the ligand binding epitopes.^19,20^ NMR-derived parameters, such as the NOE, can also be used to measure interactions of small molecules with lipids in membranes.^16^ The *R*_2_ rates measured for hyperpolarized ^19^F nuclei in a ligand that interacts with vesicle membranes depend sensitively on several dynamic processes. Our previous study indicated that these rates can be used to estimate the affinity to binding on the bilayer surface.^21^

Here, we characterize the binding of small-molecule ligands to cell membranes by using ^19^F *R*_2_ relaxometry. *R*_2_ measurements with several types of cells identify the interactions between the ligand and cell membrane proteins. Finally, we discuss the use of hyperpolarized relaxometry to study binding interactions in living cells.

## Experimental Methods

### Cell Preparation

DU4475, 4T1, and HEK293T cells were purchased from American Type Culture Collection (ATCC, Manassas, VA, USA). DU4475 human breast cancer cells or 4T1 mouse breast cancer cells were cultured in 10 mL of RPMI-1640 medium with 10% fetal bovine serum. HEK293T cells were grown in 10 mL of Dulbecco’s modified Eagle’s medium (DMEM) with 10% fetal bovine serum. Cultures were performed in a 37 °C incubator with 5% CO_2_, in T-75 flasks without shaking. The culture media were purchased from ATCC, Manassas, VA. Cells were subcultured when their density reached about 1.5 × 10^6^ cells/mL. 4T1 cells and HEK293T cells were harvested from the cell culture flask by treatment with a solution containing 0.25% trypsin and 0.53 mM EDTA (ATCC, Manassas, VA) for 2–3 min. For the experiments in which the DU4475 cells were also treated with trypsin-EDTA, the cells were centrifuged and resuspended into a trypsin-EDTA solution. After 4–6 passages from the last thawing, cells were collected for NMR experiments by centrifuging at 125*g* and resuspended in 10 mL of Dulbecco’s phosphate-buffered saline (D-PBS) buffer (ATCC, Manassas, VA). The centrifugation and resuspension were repeated three times to remove the original medium. A cell sample of about 0.7 mL was transferred to a sealed syringe and brought to the NMR room for injection into the instrument. After the NMR experiment, the cell sample that was left in the syringe was brought back to the cell culturing facility, and its cell density was counted under a Motic BA310 microscope by using Motic Images Plus 3.0 ML software. For cell counting, 15 μL of cell sample was mixed with 15 μL of trypan blue (Thermo Fisher, Waltham, MA) and added to a glass cell counting chamber.

### DNP Experiments

An aliquot of 40 mM 2-methyl-3-(5-methylsulfanyl-[1,3,4]­oxadiazol-2-yl)-6-trifluoromethyl-pyridine (ICT5040; Aobious, MA) or 4-(trifluoromethyl)­benzene-1-carboximidamide (TFBC·HCl; Maybridge, U.K.) with 15 mM 4-hydroxy-2,2,6,6-tetramethylpiperidine-1-oxyl (TEMPOL) radical (Sigma-Aldrich, St. Louis, MO) was prepared in a DMSO-*d*_6_/D_2_O (4:1 v/v) mixture. Each sample (10 μL in the gas-driven experiment and 2 μL in the liquid-driven experiment with a flow cell) was hyperpolarized for 40 min at 1.4 K in a HyperSense DNP polarizer (Oxford Instruments, Abingdon, UK) with a 3.35 T magnetic field by irradiating microwaves of 100 mW power at a frequency of 94.005 GHz. After hyperpolarization, the sample was dissolved in 4 mL of D-PBS buffer (ATCC, Manassas, VA) preheated to about 150 °C and rapidly transferred into the NMR spectrometer. To determine the signal enhancement, the sample was injected by applying forward and back-pressures of 262 and 150 psi, respectively, using a gas-driven injector.^22^ Prior to the NMR experiment, 500 ms of stabilization time was applied. For liquid-injection, the hyperpolarized solution was transferred into a 0.4 mL sample loop of a rapid injection device.^23^ A second sample loop was filled with 0.4 mL of the cell sample prior to the dissolution. To minimize cell degradation, cells were resuspended in D-PBS immediately prior to injection. The hyperpolarized sample and cells were pushed by two high-pressure syringe pumps (Models 500D and 1000D, Teledyne ISCO, Lincoln, NE), separately, into a mixer and injected into a 3D printed flow cell that was preloaded in the NMR instrument. The flow cell was printed from a clear printer resin (Anycubic). The tubing that connects the flow cell and injector was made from polyether ether ketone (PEEK). The cell waste was collected in a sealed bottle to prevent contamination of the instrument (Figure S1).

### NMR Measurements

Spectra were acquired on a 400 MHz NMR spectrometer equipped with a broadband observe (BBO) probe (Bruker Biospin, Billerica, MA). The signal acquisition took place in the flow cell, which was preloaded with the NMR probe. A closed flow path was designed to collect waste and prevent contamination from cells (Figure S1). The *R*_2_ relaxation rates were measured using a Carr–Purcell–Meiboom–Gill (CPMG) pulse sequence, *p*1 – [τ_cp_/2 – *p*2 – τ_cp_/2]_×*n*_, in a single scan. Here, *p*1 and *p*2 are π/2 and π hard pulses, and τ_cp_ is a delay of 1721.9 μs. 64 complex data points were measured between the pulses with a dwell time of 12.85 μs in each echo. After the NNR measurement, the cells were collected and inspected by a microscope and assessed for membrane integrity using trypan blue exclusion. No perfusion system was employed during the NMR measurement because of the short duration of the experiment. The data processing was carried out with Python (Python Software Foundation, https://www.python.org) by reshaping the raw data into two dimensions in accordance with the number of echoes and the number of data points acquired in each echo. Each echo was Fourier transformed after applying a sine-shaped window function and zero filling both ends of the echoes with the same amount of zeros as contained in an echo. After phase correction, the integrals of the ^19^F peaks from all spectra were fitted by using the equation *S* = *S*_0_ × exp­(–*R*_2_ × *t*) + *c* to obtain the relaxation rates.

## Results and Discussion

The transverse relaxation rates, *R*_2_, for the hyperpolarized ^19^F spins of 2-methyl-3-(5-methylsulfanyl-[1,3,4]­oxadiazol-2-yl)-6-trifluoromethyl-pyridine (ICT5040) and 4-(trifluoromethyl)­benzene-1-carboximidamide (TFBC), were measured in the presence of different types of mammalian cells (Figure 1). The cells used in the experiments were from the DU4475 human breast cancer cell line, 4T1 mouse breast cancer cells, and HEK293T human embryonic kidney cells. With the assistance of DNP, the spectra for the hyperpolarized ligands that interact with the membranes were obtained within a single scan. These experiments with living cells benefit from the fast measurement since the short signal acquisition time avoids the degradation of cells after removal from a culturing milieu. The ICT5040 molecule serves as a prototype for a drug molecule, with a reported affinity for the CXCR4 breast cancer receptor.^24^ TFBC contains an aromatic ring with a CF_3_ group similar to that of ICT5040. The binding of TFBC to trypsin protein has been studied previously,^9^ but the molecule does not have a known affinity for membrane proteins.

**Figure 1:**
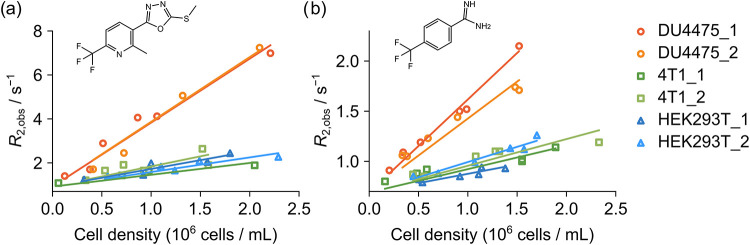
R_2_ relaxation rates measured from ^19^F hyperpolarized (a) ICT5040 and (b) TFBC in the presence of DU4475, 4T1, and HEK293T cells, respectively, at varied concentrations. The structures of the molecules are shown in each panel. Two trials were performed for each cell type. Data points in each trial were obtained by using cells from the same cell culture flask.

The DNP signal enhancement for ^19^F spins in ICT5040 and TFBC was approximately 770 and 1100-fold, respectively (ref 21 and Figure S2), measured in DNP experiments with gas-driven sample injection.^22^ At a final concentration of 33 μM, the ligands exhibited a signal-to-noise ratio on the order of 30 in the first echo of the single-scan experiments for measuring *R*_2_, as described in the Experimental section. The *R*_2_ relaxation measurements were performed by titrating the cell densities between 0 and 2.5 × 10^6^ cells/mL to study the interactions between small molecule ligands and cells. In the absence of cells, the transverse relaxation rates for ICT5040 and TFBC were *R*_2,f_ = 0.92 s^–1^ and 0.64 s^–1^. In Figures 1 and S3, the presence of each type of cell increases the observed relaxation rates, *R*_2,obs_, for both ligands. In the case of fast exchange between free and bound ligands, *R*_2,obs_ = *p* × *R*_2,b_ + (1 – *p*) × *R*_2,f_ is a population-weighted average of the *R*_2_ rates of the two forms. Here, generally, *R*_2_ of the target molecule bound to a membrane or membrane protein is larger than that for the free molecule because of slower tumbling in solution. The increase in *R*_2,obs_ therefore indicates the interaction between the small molecule ligand and the cells.

The linear increase of relaxation rates in Figure 1 is in agreement with a corresponding observation of *R*_2_ values obtained with small unilamellar vesicles.^21^ In the vesicles, the relationship can be interpreted as the initial slope of a saturation curve for weak binding to a defined number of sites on the membrane surface. Thereby, the linear dependence is valid at a limit of low lipid concentrations, whereas saturation would occur when the lipid concentration is higher than hundreds of millimolar.

To gain insight into the change in the relaxation rates observed in Figure 1, the *R*_2_ values for ICT5040 with cells can be compared to the values for vesicles (Figure 2). Here, the vesicle membranes are considered as simplified models for the cell membrane. The magenta dashed line in Figure 2 corresponds to an average of *R*_2_ relaxation observed for ICT5040 in a bilayer of a 100 nm vesicle consisting of 70 mol % 1-palmitoyl-2-oleoyl-*sn*-glycero-3-phosphocholine (POPC) and 30 mol % cholesterol in order to mimic the composition of the mammalian cell membrane.^25^ The fundamental structure of the mammalian cell membrane is the phospholipid bilayer, where the most abundant phospholipids present are phosphatidylcholines (PC).^26^ There is 20 to 50 mol % cholesterol that is distributed in mammalian membranes.^2^ A 1 μm × 1 μm area of lipid bilayer contains about 5 × 10^6^ lipid molecules.^27^ The diameters of DU4475, 4T1, and HEK293T cells were measured as 14.66 μm, 13.36 μm, and 14.36 μm, respectively (Supporting Information). Accordingly, the cell density was converted to the concentration of lipids for the solid lines in Figure 2, indicating the binding of ICT5040 to different cell types. In the double-logarithmic graph, it can be seen that *R*_2_ relaxation is more effective in cells than in the vesicles. A cell suspension with a lipid concentration that is over 10 times lower resulted in the same effect as the vesicle sample.

**Figure 2:**
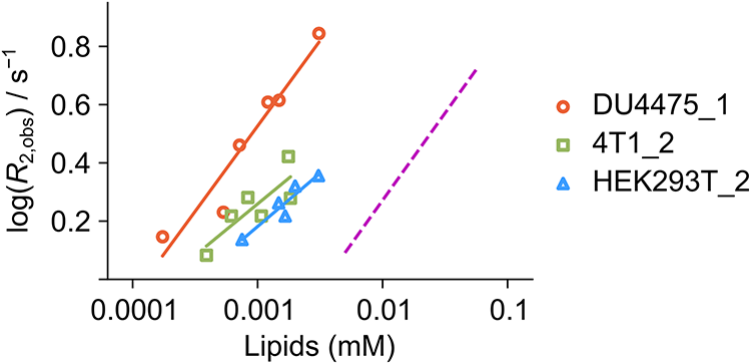
R_2_ relaxation rates measured from ^19^F hyperpolarized ICT5040 with increasing density of DU4475, 4T1, and HEK293T cells, where the cell density in Figure 1a was converted to lipid concentration (solid lines with open markers). This conversion was performed by assuming that a 1 μm × 1 μm area of lipid bilayer contains 5 × 10^6^ lipid molecules,^27^ where the cell surface area was calculated using the radius measured by ImageJ (Figure S4). The dashed line in magenta shows the R_2_ dependence for ICT5040 in the presence of 100 nm diameter POPC vesicles with 30% cholesterol.^21^

The *R*_2_ dependence on lipid concentration of cell or vesicle membranes may be understood by estimating the expected relaxation rates in both cases.^21^ An estimated correlation time τ_v_ for motions of the molecule in the membrane of a 14 μm cell based on overall tumbling of the cell and lateral diffusion of lipid molecules would be over 400 ms (Supporting Information). However, the correlation time for a small molecule that binds with a cell membrane has previously been measured in a shorter range of milliseconds.^7^ This correlation time may be due to additional membrane motions such as shape fluctuations. When τ_v_ = 3.5 ms is considered for cells, the calculated *R*_2,b_ values are 3.3 × 10^6^ s^–1^ and 2.5 × 10^6^ s^–1^ for the ICT5040 and TFBC molecules, respectively. On the other hand, lipid vesicles of 100 nm diameter have an estimated *R*_2,b_ = 1.7 × 10^4^ s^–1^ and 1.3 × 10^4^ s^–1^ for ICT5040 and TFBC, respectively.

First, the estimated relaxation is larger for ITC5040 than for TFBC because of a larger chemical shift anisotropy contribution, which is reflected in the larger relaxivity for the molecule in Figure 1. Second, the calculated *R*_2,b_ value for cells using these parameters is more than 100-fold larger than those estimated for vesicles. The *R*_2,obs_ for cells in Figure 2, indeed is larger than for vesicles, albeit the difference is less than 10-fold. The reason for this difference could be due to additional motions of the membrane that affect the *R*_2,b_ for cells, possibly resulting in different *S*_w_^2^ values or additional contributions to local motions.

In cell membranes, membrane proteins can be potential binding sites for the small molecule ligand, significantly increasing the chances of binding. Figure 3 shows the slopes of *R*_2,obs_ values from Figure 1 plotted against the cell density, indicating the relaxivity of these cells toward the ligand molecules. For both ligands, the magnitude of the *R*_2,obs_ increase obtained with DU4475 cells is about 4 times larger than that in 4T1 cells or HEK293T cells. To eliminate the variance from the cell culturing environment, the medium was removed by washing the sample three times with PBS buffer before the DNP experiments. During each washing step, the sample volume was reduced from 10 mL to 0.2–0.3 mL. Washing the cell sample three times should result in 0.001% of the original medium concentration. A two times dilution also occurred during the sample injection.^21^ Therefore, the final concentration of medium in the NMR sample is negligible, and its potential effects are not further considered. With similar *R*_2,b_ values expected for binding to lipids in different cell types of similar size, the variance in the slope of the *R*_2,obs_ curve, *i.e*., the observed relaxivity, may indicate the appearance of different amounts of protein binding sites in cell membranes.

**Figure 3:**
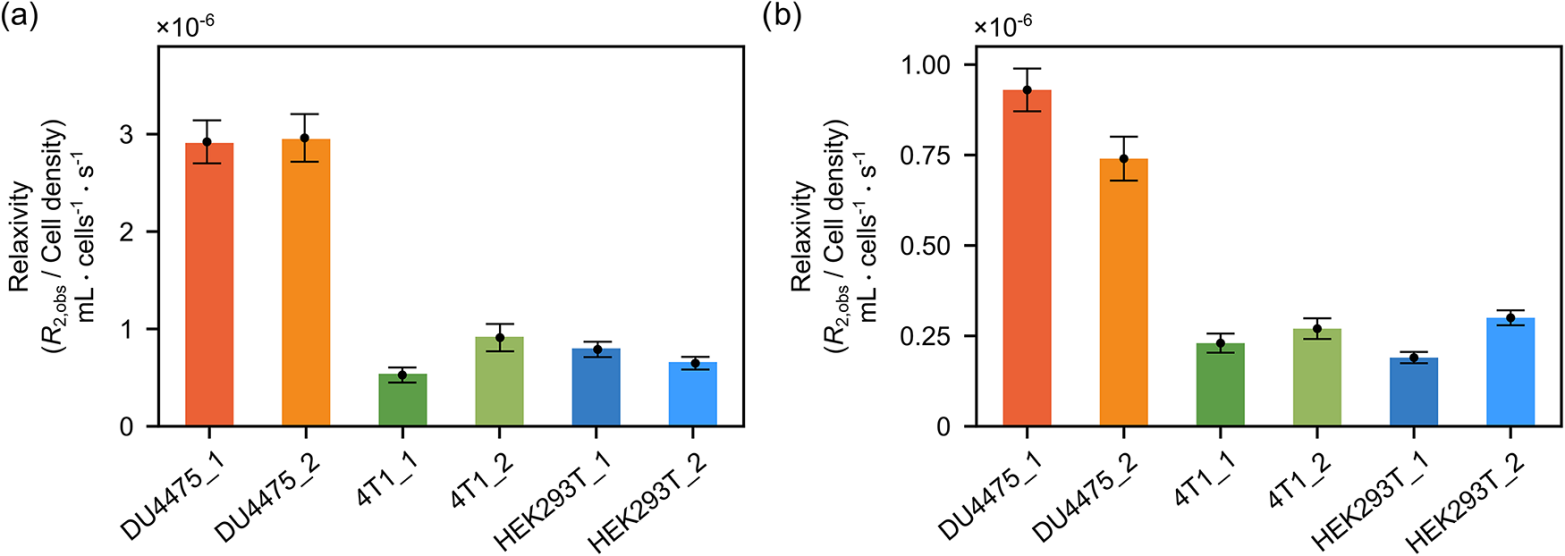
Slopes indicating relaxivity that corresponds to the *R*_2,obs_ curves measured in the presence of different types of cells, where the *R*_2,obs_ was obtained from (a) ICT5040 and (b) TFBC. Each slope was fitted from data points acquired using cells from the same cell culture flask. The error bars represent the cell concentration variances caused by the injection and the fitting errors of the *R*_2,obs_ curves.

The relaxivity for TFBC is less than for ICT5040. This difference is probably caused by the different binding affinities of ICT5040 and TFBC. However, the magnitude of *R*_2,obs_ increase obtained in the presence of DU4475 cells is about 4 times larger than that with other cells, both for ICT5040 and for TFBC. The different relaxivities lead to the possible conclusion that the number of proteins providing binding sites is more abundant in the membrane of DU4475 cells rather than the other two types of cells.

The DU4475 cells are cultured in suspension, whereas 4T1 and HEK293T cells adhere to a surface. The procedure that detaches the adherent cells from the cell culture flask before measurement may affect the density of cell surface proteins. When the DU4475 cells are also treated with trypsin, digesting proteins in the membrane, the resulting relaxivity is smaller compared to untreated DU4475 cells (Figure 4a). Since trypsinization does not affect the size of cells or the *R*_2,b_, this *R*_2,obs_ difference should be caused by the reduction in protein species, indicating that ligand-membrane protein interactions play a significant role in the observed transverse relaxation rates of the ligand.

**Figure 4:**
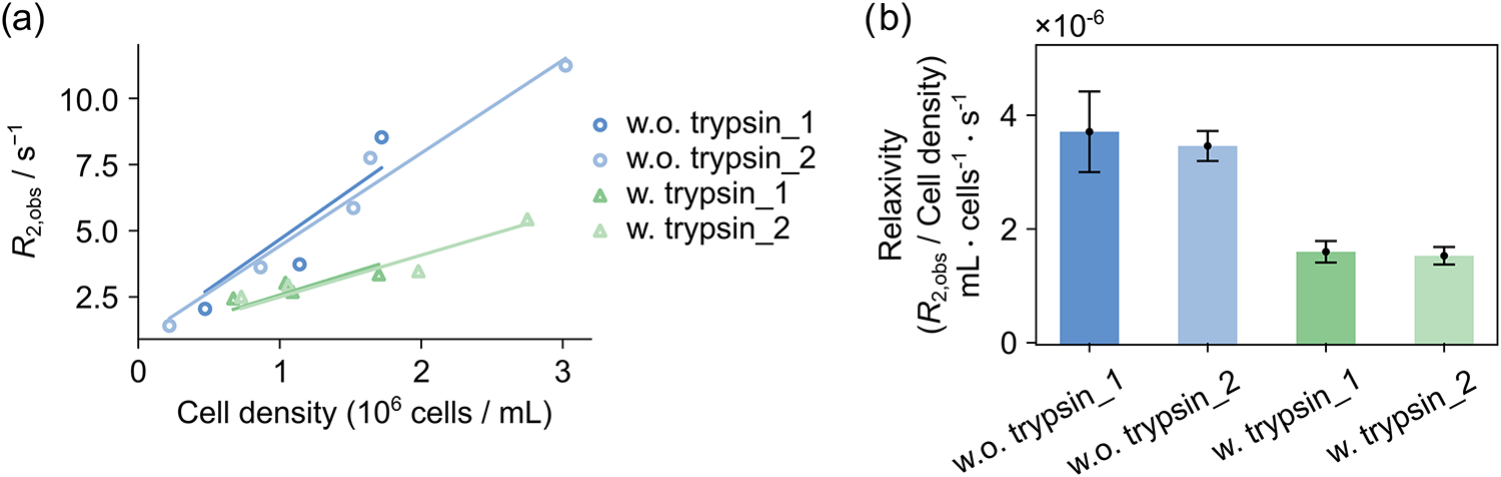
(a) R_2_ relaxation rates obtained from ^19^F hyperpolarized ICT5040 in the presence of DU4475 cells treated with or without trypsin-EDTA before the measurement. The procedure for the experiments without trypsin treatment was the same as that for the experiments included in Figure 1. Two trials of experiments with or without trypsinization were performed to show the reproducibility. Data points in each of the trials were obtained using cells from the same cell culture flask. (b) Corresponding slopes for *R*_2,obs_ curves indicating relaxivity of the cells toward ICT5040, with error bars showing the errors from cell concentration variances and curve fittings. w.o. and w. refer to without and with, respectively.

The addition of a competitive binder, AMD3100, or an antibody targeting CXCR4, together with hyperpolarized ICT5040, did not result in a significant change in observed *R*_2_ rates (Figure S5). Therefore, we conclude that binding to membrane proteins significantly affects the relaxation rate of the observed small molecule ligand. Both specific and nonspecific interactions can contribute to the relaxation change, whereby the DU4475 cells with AMD3100 or CD184 monoclonal antibody (12G5) did not result in a conclusive observation of specific binding.

Identifying the binding of a ligand molecule to cell surface proteins in living cells can be difficult due to the complexity of the cell environment. The data described demonstrate that an NMR-based spin relaxation measurement can detect ligand–membrane protein interactions in a short time period. The ability to acquire data in a single scan using nuclear spin hyperpolarization reduces the risk of damaging the cells during the experiment. The *R*_2_ relaxation approach has the advantage that the cells exhibit a long correlation time and fast *R*_2,b_ that makes the observed relaxation rate sensitive to the binding. With the assistance of hyperpolarization, the ligand concentration can be significantly reduced compared to that in other NMR experiments.

## Conclusions

The transverse relaxation rate, *R*_2_, was measured for small molecule ligands that interact with cells to study the interactions between ligands, membranes, and membrane proteins. The NMR signal was enhanced by hyperpolarization and can be acquired in a single scan. *R*_2_ relaxation parameters in cells were significantly different from those obtained in the presence of the model membranes in vesicles. The comparison suggests that the linear dependence of observed *R*_2_ on cell density can be explained by ligand–membrane binding, with a significant contribution of binding to membrane proteins. The interactions between ligands and cell membrane proteins are reflected by the different magnitudes of the *R*_2,obs_ increase observed from experiments with different cell types. When some membrane proteins are removed with trypsin, a smaller *R*_2_ increase indicates that less binding occurred on the cell surface. These results suggest that model membranes in many cases may not be sufficient for binding studies. A method based on *R*_2_ measurements from hyperpolarized ligands may further be applicable for the measurement of interactions with membrane proteins in drug screening and biophysical studies.

## Supplementary Material



## Notes

Instrumentation, hyperpolarized NMR spectra, *R*_2_ relaxation curves, *R*_2_ relaxation rates, cell size measurements, relaxation calculations, and competitive binding *R*_2_ experiments (PDF)
