# Supplementary material for: Characterization of Cell–Surface Interactions of Ligands Using 19F NMR and DNP Hyperpolarization
Source: Anal Chem. 2025 Dec 26;98(1):317–22. doi: 10.1021/acs.analchem.5c04644 (PMC12809643; doi:10.1021/acs.analchem.5c04644)
Supplement: Supplementary file 1 [file ac5c04644_si_001.pdf]

Supporting Information

Characterization of Cell-Surface Interactions of Ligands using ^{19}F NMR and DNP Hyperpolarization

Chang Qi, Nirmalya Pradhan and Christian Hilty*

Chemistry Department, Texas A&M University, College Station, TX 77843, USA

*email: chilty@tamu.edu

Table of Contents

Instrumentation........ S2
Hyperpolarized NMR Spectra........ S2
R_2 Relaxation Curves........ S3
R_2 Relaxation Rates........ S3
Cell Size Measurements........ S6
Relaxation Calculations........ S7
Competitive Binding R_2 Experiments........ S8
References........ S9

Instrumentation

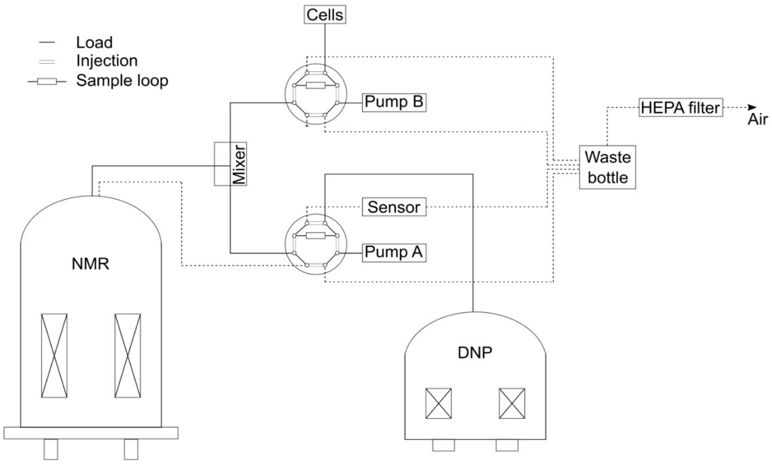

Figure S1. *Instrumentation for the DNP experiments with cells. Solid lines indicate tubing for the transfer of cells and the hyperpolarized ligand into the flow cell inside the NMR. Dashed lines indicate the waste collection of the sample that flowed out of the NMR spectrometer, the cell sample, and the hyperpolarized ligand sample by a sealed waste bottle with a HEPA filter. The whole system is closed to prevent aerosols containing cells to be released into the room atmosphere.*

In the experiment that mixed the hyperpolarized ligand with cells, cell samples were preloaded in a sample loop when the injector was in load mode. After hyperpolarization, the ligand filled the bottom sample loop and triggered the optical sensor. Both injectors were switched from load mode into injection mode at different times. The timing parameters for switching of the injector valves were optimized to achieve the highest NMR signal enhancement obtained in the flow cell. Waste that flowed out of the NMR flow cell was collected by a sealed waste bottle with a HEPA filter connected.

Hyperpolarized NMR Spectra

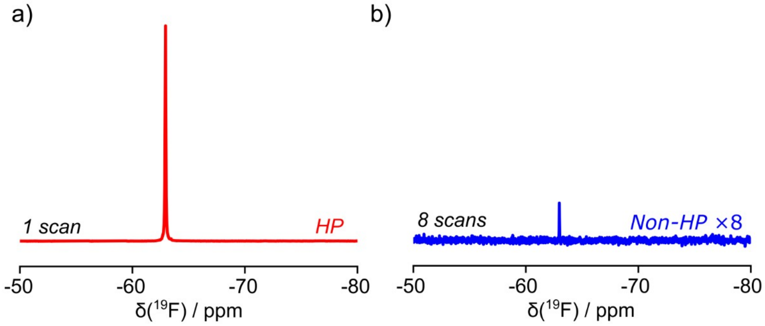

Figure S2. *a) ^{19}F hyperpolarized NMR spectra of 0.32 mM TFBC measured after transfer of the solution to the NMR spectrometer using gas-driven injection (HP, red). b) Non-hyperpolarized ^{19}F NMR spectrum of 0.32 mM TFBC (Non-HP, blue). The spectrum was measured of the same solution used to perform the hyperpolarized experiment. It was measured with 8 scans and additionally scaled up by 8-fold.*

The ^{19}F hyperpolarization enhancement for TFBC was measured by comparing the integral of the hyperpolarized signal to the signal measured from the same sample after hyperpolarization had decayed. The hyperpolarized spectrum was acquired by injecting the hyperpolarized solution into an NMR tube inside the spectrometer using a gas-driven injector. The gas-driven injection was used instead of the liquid driven injection into a flow cell to ensure that the two signals were measured from the same solution without the possibility for movement between the measurements. The calculated signal enhancement for TFBC was 1100.

R_2 Relaxation Curves

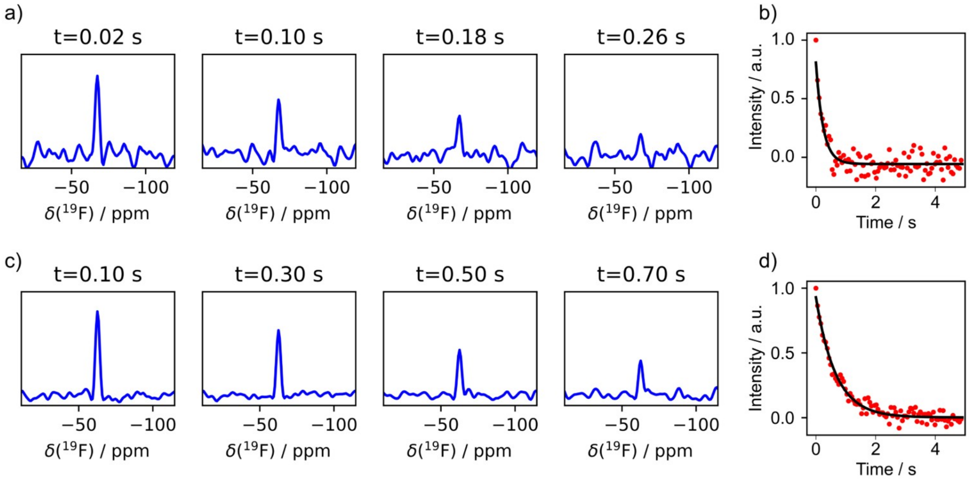

Figure S3. *Spectra obtained in the CPMG experiments for hyperpolarized fluorine spins of 33 µM a) ICT5040 and c) TFBC in the presence of 10^6 cells/mL DU4475 cells. The R_2 decays for ICT5040 and TFBC were plotted in b) and d). The fitted R_2 are 4.13 s^{-1} and 1.52 s^{-1}, respectively.*

R_2 Relaxation Rates

Table S1. *Relaxation rates of hyperpolarized ICT5040 in the presence of DU4475 cells with their density indicated. Cell density was calculated by counting cells after the DNP experiment. Error ranges from R_2 fitting are indicated. Two repetitions were performed.*

Trial 1		Trial 2	
10^6 cells/ mL	R_2 (s^{-1})	10^6 cells/ mL	R_2 (s^{-1})
0.41	1.71 ± 0.02	0.12	1.40 ± 0.02
0.72	2.45 ± 0.04	0.38	1.70 ± 0.02
1.32	5.06 ± 0.09	0.51	2.89 ± 0.06
2.1	7.24 ± 0.21	0.86	4.06 ± 0.08
		1.06	4.12 ± 0.09
		2.21	6.99 ± 0.16

Table S2. *Relaxation rates of hyperpolarized ICT5040 in the presence of 4T1 cells with their density indicated. Cell density was calculated by counting cells after the DNP experiment. Error ranges from R_2 fitting are indicated. Two repetitions were performed.*

Trial 1		Trial 2	
10^6 cells/ mL	R_2 (s^{-1})	10^6 cells/ mL	R_2 (s^{-1})
0.06	1.08 ± 0.02	0.34	1.21 ± 0.07
0.704	1.43 ± 0.02	0.53	1.65 ± 0.02
0.976	1.62 ± 0.01	0.92	1.65 ± 0.03
2.05	1.89 ± 0.02	1.58	1.90 ± 0.03
		1.52	2.64 ± 0.02
		0.72	1.91 ± 0.05

Table S3. *Relaxation rates of hyperpolarized ICT5040 in the presence of HEK293T cells with their density indicated. Cell density was calculated by counting cells after the DNP experiment. Error ranges from R_2 fitting are indicated. Two repetitions were performed.*

Trial 1		Trial 2	
10^6 cells/ mL	R_2 (s^{-1})	10^6 cells/ mL	R_2 (s^{-1})
0.06	1.08 ± 0.02	0.34	1.21 ± 0.07
0.70	1.43 ± 0.02	0.53	1.65 ± 0.02
0.98	1.62 ± 0.01	0.92	1.65 ± 0.03
2.05	1.89 ± 0.02	1.58	1.90 ± 0.03
		1.52	2.64 ± 0.02
		0.72	1.91 ± 0.05

Table S4. *Relaxation rates of hyperpolarized TFBC in the presence of DU4475 cells with their density indicated. Cell density was calculated by counting cells after the DNP experiment. Error ranges from R_2 fitting are indicated. Two repetitions were performed.*

Trial 1		Trial 2	
10^6 cells/ mL	R_2 (s^{-1})	10^6 cells/ mL	R_2 (s^{-1})
0.20	0.91 ± 0.01	1.52	1.70 ± 0.01
0.92	1.50 ± 0.01	1.48	1.75 ± 0.01
1.99	1.53 ± 0.01	0.34	1.06 ± 0.01
1.52	2.14 ± 0.02	0.90	1.44 ± 0.01

0.34	1.09 ± 0.01	0.60	1.22 ± 0.01
0.52	1.18 ± 0.01	0.38	1.05 ± 0.01

Table S5. *Relaxation rates of hyperpolarized TFBC in the presence of 4T1 cells with their density indicated. Cell density was calculated by counting cells after the DNP experiment. Error ranges from* R_2 *fitting are indicated. Two repetitions were performed.*

Trial 1		Trial 2	
10^6 cells/ mL	R_2 (s^{-1})	10^6 cells/ mL	R_2 (s^{-1})
1.89	1.14 ± 0.01	2.33	1.19 ± 0.01
1.55	1.03 ± 0.01	1.28	1.10 ± 0.01
1.55	1.00 ± 0.01	1.32	1.10 ± 0.01
0.58	0.92 ± 0.01	1.08	1.05 ± 0.01
0.48	0.88 ± 0.01	0.44	0.87 ± 0.01
0.16	0.80 ± 0.00	0.56	0.85 ± 0.01

Table S6. *Relaxation rates of hyperpolarized TFBC in the presence of HEK293T cells with their density indicated. Cell density was calculated by counting cells after the DNP experiment. Error ranges from* R_2 *fitting are indicated. Two repetitions were performed.*

Trial 1		Trial 2	
10^6 cells/ mL	R_2 (s^{-1})	10^6 cells/ mL	R_2 (s^{-1})
1.38	0.93 ± 0.01	1.57	1.12 ± 0.01
1.14	0.94 ± 0.01	1.70	1.26 ± 0.01
1.11	0.87 ± 0.01	1.43	1.13 ± 0.01
0.92	0.85 ± 0.01	1.07	0.99 ± 0.01
0.50	0.82 ± 0.01	0.70	0.88 ± 0.01
0.54	0.79 ± 0.01	0.45	0.85 ± 0.01

Cell Size Measurements

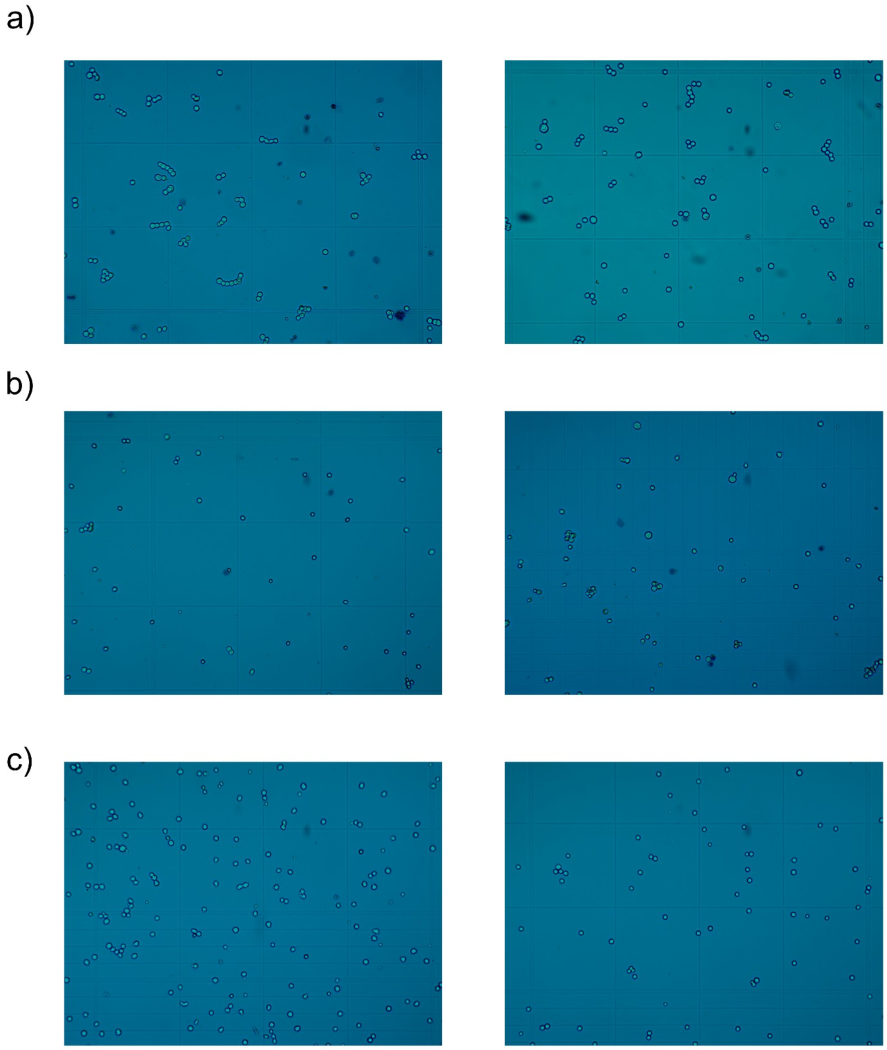

Figure S4. *Cell images for a) DU4475, b) 4T1, and c) HEK293T cells obtained using a microscope with a 40× objective lens.*

The cell size was measured by determining the area of all cells in each image using ImageJ and calculating the averaged area per cell. The diameter of the cells in Table 7 is the averaged result obtained from two images for each cell types shown in Figure S4.

The number of lipid molecules per cell was estimated assuming that a 1 µm × 1 µm area of lipid bilayer contains $5{\cdot}10^6$ lipid molecules.[1] The resulting equation is

$$n = 5 \cdot 10^6 \,\text{lipids}\, \mu\text{m}^{-1} \;\; \times \pi \left(\frac{d}{2}\right)^2 \qquad \text{(S1)}$$

where *d* is the cell diameter included in Table S7.

Table S7. *Cell diameters measured with ImageJ and the estimated number of lipid molecules contained in each cell membrane for three cell types*

Cell type	Cell diameter *d* [μm]	(10^8) lipids / cell
DU4475	14.66 ± 0.31	8.44 ± 0.18
4T1	13.36 ± 0.16	7.01 ± 0.09
HEK293T	14.36 ± 0.16	8.09 ± 0.09

Relaxation Calculations

The R_2 relaxation rate of a molecule bound to the membrane was estimated loosely following the assumptions and equations in ref.[2] The $R_{2,b} = R_{2,HF(DD)} + 2R_{2,FF(DD)} + R_{2(CSA)}$ includes the dipole-dipole (DD) interaction, $R_{2,HF(DD)}$, between fluoromethyl and the nearest proton, in addition to $2R_{2,FF(DD)}$ as intra-fluoromethyl relaxation. The anisotropic chemical shielding (CSA) relaxation contribution is $R_{2(CSA)}$[3]

$$R_{2,HF(DD)}=\frac{1}{20}\left(\frac{\mu_0\hbar\,\gamma_H\,\gamma_F}{4\pi r_{HF}^3}\right)^2\{4J(0)+J(\omega_H-\omega_F)+3J(\omega_F)+6J(\omega_H)+6J(\omega_H+\omega_F)\} \qquad \text{(S4)}$$

$$R_{2,FF(DD)}=\frac{3}{20}\left(\frac{\mu_0\hbar\,\gamma_F^2}{4\pi r_{FF}^3}\right)^2\{3J(0)+5J(\omega_F)+2J(2\omega_F)\} \qquad \text{(S5)}$$

$$R_{2(CSA)}=\frac{1}{45}(\gamma_F B_0\Delta\sigma)^2[4J(0)+3J(\omega_F)] \qquad \text{(S6)}$$

In these equations, $\mu_0 = 4\pi\cdot10^{-7}$ Hm^{-1}, $\hbar = 1.055\cdot10^{-34}$ Js, $\gamma_H = 2.68\cdot10^8$ s^{-1}T^{-1}, $\gamma_F = 2.52\cdot10^8$ s^{-1}T^{-1}, $B_0 = 9.4$ T, and $\omega = 2\pi\cdot\gamma$. The distance between the ^{19}F to ^{1}H spins and ^{19}F to ^{19}F spins are r_{HF} *and* r_{FF} *respectively*, and the chemical shift anisotropy is $\Delta\sigma$ (see below). In the spectral density for intramethyl relaxation, cross-correlation is not considered.

Local motions of the molecule can be accounted for using the extended model free treatment.[4,5] The spectral density is

$$J_{ext}(\omega)=S_w^2S_r^2\frac{\tau_v}{1+(\omega\tau_v)^2}+S_w^2(1-S_r^2)\frac{\tau_r'}{1+(\omega\tau_r')^2}+(1-S_w^2)\frac{\tau_w'}{1+(\omega\tau_w')^2} \qquad \text{(S7)}$$

The rotational correlation time τ_c can be calculated from the Stokes-Einstein equation

$$\tau_c=\frac{4\pi\eta R^3}{3kT} \qquad \text{(S8)}$$

where $\eta = 8.9\cdot10^{-4}$ Pa·s is the dynamic viscosity of the solvent, *i.e.* water. R is the average of the radii of vesicle or cells, $k = 1.38\cdot10^{-23}$ J K^{-1} is Boltzman's constant and $T = 298$ K is the temperature. The radii of the vesicle and cell are 50 nm and about 7 μm, respectively. The τ are the correlation times for motions described below, and S are the corresponding order parameters.

For a molecule that laterally diffuses along the curved shell of the membrane, the transverse diffusion contributes to the effective correlation time

$$\tau_v = \left(\tau_c^{-1} + \tau_t^{-1}\right)^{-1} = \left(\frac{3kT}{4\pi\eta R^3} + \frac{6D}{R^2}\right)^{-1} \tag{S9}$$

D is the transverse diffusion coefficient, which for lipids from POPC vesicles is $1.9\cdot10^{-11}$ m^2s^{-1}.[6] The diffusion coefficient for the small molecule in a cell membrane is assumed to be the same. For cells of 14 µm diameter, the calculated τ_v = 429 ms. For 100 nm vesicles, τ_v = 18 µs.

The molecule is considered to exhibit a local motion, which may be modeled as a "wobbling" motion, which results in an effective correlation time of

$$\tau_w{}' = \left(\tau_v^{-1} + \tau_w^{-1}\right)^{-1} \tag{S10}$$

The CF_3 rotation contributes with an effective corelation time of

$$\tau_r{}' = \left(\tau_v^{-1} + \tau_r^{-1}\right)^{-1} \tag{S11}$$

Here, S_w^2 represent the order parameters for the motion of the entire small molecule in the membrane, which may be described as a "wobble-in-cone" motion. S_r^2 describe the rotational order parameter for CF_3 group.

For membrane binding, he local motion of ligand movement with a typical diffusion coefficient $D_w = 1/(6\tau_w) \sim 10^7 - 10^9$ and a wobble order parameter, $S_w^2 = 0.4$ were considered in the calculation.[7,8] The fast rotation of the CF_3 group, $\tau_r = 10^{-11}$ s with a rotational order parameter, $S_{r,HF}^2 = 0.53$ and $S_{r,FF}^2 = 0.25$ were also included.[9] The order parameter was calculated using an ensemble average of random traces generated from jumps between three sites with time.[10,11] The ^{19}F and the closest averaged ^1H at a distance of 3.01 Å and 3.02 Å for the ICT5040 and TFBC molecules, respectively, were included in dipole-dipole relaxation. Additionally, r_{FF} = 2.25 Å was included in dipole-dipole relaxation for both the compounds. In addition, the calculated chemical shift anisotropy[12] of -56 ppm for ICT5040 and -45 ppm for TFBC were used in the calculation.

For binding to the cell membrane with τ_v = 429 ms, the calculated $R_{2,b}$ value is $4\cdot10^8$ s^{-1} and $3\cdot10^8$ s^{-1} for ICT5040 and TFBC molecule, respectively. When τ_v = 3.5 ms is considered for cells, the calculated $R_{2,b}$ value is $3.3\cdot10^6$ s^{-1} and $2.5\cdot10^6$ s^{-1} for ICT5040 and TFBC molecule, respectively. For a 100 nm vesicle, the estimated $R_{2,b} = 1.7\cdot10^4$ s^{-1} and $1.3\cdot10^4$ s^{-1} for ICT5040 and TFBC molecule respectively with the above mentioned same parameter.

Competitive Binding R_2 Experiments

In the competitive binding experiment, 30 µL 2 mM AMD3100 (Sigma, St. Louis, MO) or 25 µL (1.25 µg) CD184 (CXCR4) monoclonal antibody (12G5) (Fisher Scientific, Hampton, NH) was mixed with DU4475 cells. After incubation for half an hour to achieve equilibrium, the sample was centrifuged and

resuspended in a D-PBS buffer (ATCC, Manassas, VA) as described in the main text. This step was repeated three times to remove the original medium. About 0.7 mL cell sample mixed with AMD3100 or antibody was obtained and loaded into the syringe for the NMR experiment. Antibody binding to DU4475 cells was verified using a flow cytometric assay.

AMD3100 is an inhibitor for the CXCR4 cell surface receptor (IC_{50} = 44 nM).[13] In the competitive binding experiment, the hyperpolarized ligand should be displaced by the stronger binder AMD3100 or antibody and exhibits less $R_{2,obs}$ increase. However, here the relaxation rates was not significantly affected by the presence of a stronger binder. Therefore, the specific binding of the ligand ICT5040 is not conclusively identified by these competitive binding experiments.

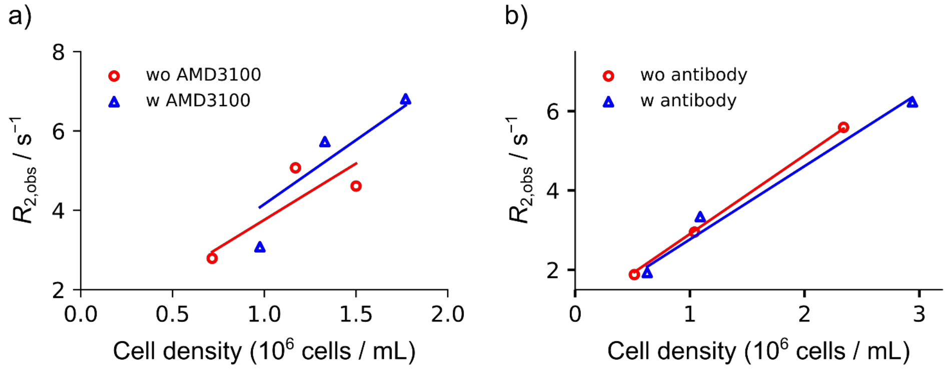

Figure S5. *R_2 obtained in the competitive binding experiments for the ligand ICT5040 in the presence of DU4475 human breast cancer cells with and without a) 30 µL 2 mM AMD3100, or with and without b) 25 µL CXCR4 antibody. wo and w refers to without and with, respectively.*

References

(1) Alberts, B.; Johnson, A.; Lewis, J.; Raff, M.; Roberts, K.; Walter, P. The Lipid Bilayer. In *Molecular Biology of the Cell. 4th edition*; Garland Science, 2002.

(2) Qi, C.; Pradhan, N.; Hilty, C. Quantification of Ligand-Membrane Interactions Using DNP-NMR Relaxometry. *Submitted*.

(3) Cavanagh, J.; Fairbrother, Wayne. J.; Palmer III, A. G.; Rance, Mark; Skelton, Nicholas J. *Protein NMR Spectroscopy: Principles and Practice*, 2nd Ed.; Elsevier, Acad. Press: Amsterdam, 2007.

(4) Lipari, G.; Szabo, A. Model-Free Approach to the Interpretation of Nuclear Magnetic Resonance Relaxation in Macromolecules. 1. Theory and Range of Validity. *J. Am. Chem. Soc.* **1982**, *104* (17), 4546–4559. https://doi.org/10.1021/ja00381a009.

(5) Clore, G. M.; Driscoll, P. C.; Wingfield, P. T.; Gronenborn, A. M. Analysis of the Backbone Dynamics of Interleukin-1.Beta. Using Two-Dimensional Inverse Detected Heteronuclear Nitrogen-15-Proton NMR Spectroscopy. *Biochemistry* **1990**, *29* (32), 7387–7401. https://doi.org/10.1021/bi00484a006.

(6) Gaede, H. C.; Gawrisch, K. Lateral Diffusion Rates of Lipid, Water, and a Hydrophobic Drug in a Multilamellar Liposome. *Biophys. J.* **2003**, *85* (3), 1734–1740. https://doi.org/10.1016/s0006-3495(03)74603-7.

(7) Lipari, G.; Szabo, A. Effect of Librational Motion on Fluorescence Depolarization and Nuclear Magnetic Resonance Relaxation in Macromolecules and Membranes. *Biophys. J.* **1980**, *30* (3), 489–506.
(8) Steele, H. B. B.; Sydor, M. J.; Anderson, D. S.; Holian, A.; Ross, J. B. A. Using Time-Resolved Fluorescence Anisotropy of Di-4-ANEPPDHQ and F2N12S to Analyze Lipid Packing Dynamics in Model Systems. *J. Fluoresc.* **2019**, *29* (2), 347–352. https://doi.org/10.1007/s10895-019-02363-7.
(9) Richarz, R.; Nagayama, K.; Wüthrich, K. Carbon-13 Nuclear Magnetic Resonance Relaxation Studies of Internal Mobility of the Polypeptide Chain in Basic Pancreatic Trypsin Inhibitor and a Selectively Reduced Analog. *Biochemistry* **1980**, *19* (23), 5189–5196. https://doi.org/10.1021/bi00564a006.
(10) Singer, P. M.; Asthagiri, D.; Chapman, W. G.; Hirasaki, G. J. Molecular Dynamics Simulations of NMR Relaxation and Diffusion of Bulk Hydrocarbons and Water. *J. Magn. Reson.* **2017**, *277*, 15–24. https://doi.org/10.1016/j.jmr.2017.02.001.
(11) Tropp, J. Dipolar Relaxation and Nuclear Overhauser Effects in Nonrigid Molecules: The Effect of Fluctuating Internuclear Distances. *J. Chem. Phys.* **1980**, *72* (11), 6035–6043. https://doi.org/10.1063/1.439059.
(12) Rüdisser, S. H.; Goldberg, N.; Ebert, M.-O.; Kovacs, H.; Gossert, A. D. Efficient Affinity Ranking of Fluorinated Ligands by 19F NMR: CSAR and FastCSAR. *J. Biomol. NMR* **2020**, *74* (10–11), 579–594. https://doi.org/10.1007/s10858-020-00325-x.
(13) Gerlach, L. O.; Skerlj, R. T.; Bridger, G. J.; Schwartz, T. W. Molecular Interactions of Cyclam and Bicyclam Non-Peptide Antagonists with the CXCR4 Chemokine Receptor. *J. Biol. Chem.* **2001**, *276* (17), 14153–14160. https://doi.org/10.1074/jbc.M010429200.
